# Supplementary material for: CRISPRi–TnSeq maps genome-wide interactions between essential and non-essential genes in bacteria
Source: Nat Microbiol. 2024 Jul 19;9(9):2395–409. doi: 10.1038/s41564-024-01759-x (PMC11371651; doi:10.1038/s41564-024-01759-x)
Supplement: Supplementary file 1 — Reporting Summary [file 41564_2024_1759_MOESM1_ESM.pdf]

nature portfolio

Corresponding author(s): Dr. Tim van Opijnen

Last updated by author(s): May 20, 2024

Reporting Summary

Nature Portfolio wishes to improve the reproducibility of the work that we publish. This form provides structure for consistency and transparency in reporting. For further information on Nature Portfolio policies, see our Editorial Policies and the Editorial Policy Checklist.

Statistics

For all statistical analyses, confirm that the following items are present in the figure legend, table legend, main text, or Methods section.

n/a	Confirmed	
☐	☒	The exact sample size (n) for each experimental group/condition, given as a discrete number and unit of measurement
☐	☒	A statement on whether measurements were taken from distinct samples or whether the same sample was measured repeatedly
☐	☒	The statistical test(s) used AND whether they are one- or two-sided<br>*Only common tests should be described solely by name; describe more complex techniques in the Methods section.*
☐	☒	A description of all covariates tested
☐	☒	A description of any assumptions or corrections, such as tests of normality and adjustment for multiple comparisons
☐	☒	A full description of the statistical parameters including central tendency (e.g. means) or other basic estimates (e.g. regression coefficient) AND variation (e.g. standard deviation) or associated estimates of uncertainty (e.g. confidence intervals)
☐	☒	For null hypothesis testing, the test statistic (e.g. F, t, r) with confidence intervals, effect sizes, degrees of freedom and P value noted<br>*Give P values as exact values whenever suitable.*
☒	☐	For Bayesian analysis, information on the choice of priors and Markov chain Monte Carlo settings
☐	☒	For hierarchical and complex designs, identification of the appropriate level for tests and full reporting of outcomes
☐	☒	Estimates of effect sizes (e.g. Cohen's d, Pearson's r), indicating how they were calculated

Our web collection on statistics for biologists contains articles on many of the points above.

Software and code

Policy information about availability of computer code

Data collection	CRISPRi-TnSeq NGS data was collected on NextSeq sequencing platform (illumina). Growth data were collected on BioSpa spectrophotometer (Agilent). Fluorescence signal was recorded on Tecan Spectrometer. Microscopy images were captured on Olympus IX83 (Olympus) or Deltavision Elite (GE Healthcare).
Data analysis	CRISPRi-TnSeq NGS data was analyzed by Aerobio sequencing analyses platform, which is publicly available. Open source platform "Vega-Lite" and commercial software "GraphPad Prism 6" were used for fitness and growth data presentation, respectively. Microscopy images were processed by open software "Image J" and analyzed by python package "MOMIA" and "Scikit-image", available on Gitgub. 3D-Structural Illumination Microscopy images were processed by commercial software "SoftWoRx (GE Healthcare)". Gene set enrichment analysis (GSEA) was performed using open source GSEA 4.1.0 platform. Pathway analyses were performed using open source web-platform BioCyc. Genetic and chemical genetic interaction datasets were visualized with open source software Cytoscape 3.8.0.

For manuscripts utilizing custom algorithms or software that are central to the research but not yet described in published literature, software must be made available to editors and reviewers. We strongly encourage code deposition in a community repository (e.g. GitHub). See the Nature Portfolio guidelines for submitting code & software for further information.

Data

Policy information about availability of data

All manuscripts must include a data availability statement. This statement should provide the following information, where applicable:

- Accession codes, unique identifiers, or web links for publicly available datasets
- A description of any restrictions on data availability
- For clinical datasets or third party data, please ensure that the statement adheres to our policy

All sequence data can be found in the NCBI Sequence Read Archive under the BioProject: PRJNA813307. NCBI Reference Genome Sequence file NC_008533.2 was used for the analysis of CRISPRi-TnSeq data. Source data for the figures are available in the corresponding source data tables.

Research involving human participants, their data, or biological material

Policy information about studies with human participants or human data. See also policy information about sex, gender (identity/presentation), and sexual orientation and race, ethnicity and racism.

Reporting on sex and gender	This is a study on bacterial genetic interaction mapping, so sex is not relevant.
Reporting on race, ethnicity, or other socially relevant groupings	This is a study on bacterial genetic interaction mapping, so race is not relevant.
Population characteristics	This is a study on bacterial genetic interaction mapping, so population characteristics is not relevant.
Recruitment	This is a study on bacterial genetic interaction mapping, so recruitment is not relevant.
Ethics oversight	This is a study on bacterial genetic interaction mapping, so ethics is not relevant.

Note that full information on the approval of the study protocol must also be provided in the manuscript.

Field-specific reporting

Please select the one below that is the best fit for your research. If you are not sure, read the appropriate sections before making your selection.

☒ Life sciences ☐ Behavioural & social sciences ☐ Ecological, evolutionary & environmental sciences

For a reference copy of the document with all sections, see nature.com/documents/nr-reporting-summary-flat.pdf

Life sciences study design

All studies must disclose on these points even when the disclosure is negative.

Sample size	6 samples/condition. In previous articles we demonstrated that 6 samples per condition provides data with statistical rigor (van Opijnen et al., Nat Methods 2009; van Opijnen et al., Genome Res. 2012; van Opijnen et al., Curr. Protoc. Mol. Biol. 2014).
Data exclusions	When growth inhibition is severe Tn-Seq identifies non-specific interactions, therefore we excluded those samples which is described in the Materials section.
Replication	Each CRISPRi-TnSeq experiment was performed with at least 2 different IPTG concentrations and with 6 individual libraries. Growth and gene expression experiments were performed with 3 biological and 3 technical replicates. Independent repetition of all experiments produced similar results.
Randomization	A random genetic interaction dataset was created by permutation to compare with the experimentally observed dataset. This comparison shows that observed dataset is enriched with negative genetic interactions.
Blinding	While blinding is not relevant, extensive statistics and validation experiments all but guarantee the robustness of the dataset.

Reporting for specific materials, systems and methods

We require information from authors about some types of materials, experimental systems and methods used in many studies. Here, indicate whether each material, system or method listed is relevant to your study. If you are not sure if a list item applies to your research, read the appropriate section before selecting a response.

Materials & experimental systems

n/a	Involved in the study
☐	☒ Antibodies
☒	☐ Eukaryotic cell lines
☒	☐ Palaeontology and archaeology
☒	☐ Animals and other organisms
☒	☐ Clinical data
☒	☐ Dual use research of concern
☒	☐ Plants

Methods

n/a	Involved in the study
☒	☐ ChIP-seq
☒	☐ Flow cytometry
☒	☐ MRI-based neuroimaging

Antibodies

Antibodies used: Anti-Cyclic-di-AMP antibody

Validation: The above antibody was purchased with ELISA assay kit of Cayman chemicals, catalog number 501960. Details can be found on company's website "https://www.caymanchem.com/product/501960/cyclic-di-amp-elisa-kit".

Plants

Seed stocks: n.a.

Novel plant genotypes: n.a.

Authentication: n.a.
